# Detection and Identification of Old World *Leishmania* by High Resolution Melt Analysis

**DOI:** 10.1371/journal.pntd.0000581

**Published:** 2010-01-12

**Authors:** Dalit Talmi-Frank, Abedelmajeed Nasereddin, Lionel F. Schnur, Gabriele Schönian, Seray Özensoy Töz, Charles L. Jaffe, Gad Baneth

**Affiliations:** 1 School of Veterinary Medicine, Hebrew University, Rehovot, Israel; 2 Kuvin Centre for the Study of Tropical and Infectious Diseases, IMRIC, Hebrew University-Hadassah Medical School, Jerusalem, Israel; 3 Al-Quds University, Leishmaniasis Research Center, Abu-Deis, The Palestinian Authority; 4 Institute of Microbiology and Hygiene, Charité Universitätsmedizin Berlin, Berlin, Germany; 5 Department of Parasitology, The Ege University Medical School, Izmir, Turkey; Institut Pasteur de Tunis, Tunisia

## Abstract

**Background:**

Three major forms of human disease, cutaneous leishmaniasis, visceral leishmaniasis and mucocutaneous leishmaniasis, are caused by several leishmanial species whose geographic distribution frequently overlaps. These *Leishmania* species have diverse reservoir hosts, sand fly vectors and transmission patterns. In the Old World, the main parasite species responsible for leishmaniasis are *Leishmania infantum*, *L. donovani*, *L. tropica*, *L. aethiopica* and *L. major*. Accurate, rapid and sensitive diagnostic and identification procedures are crucial for the detection of infection and characterization of the causative leishmanial species, in order to provide accurate treatment, precise prognosis and appropriate public health control measures.

**Methods/Principal Findings:**

High resolution melt analysis of a real time PCR product from the Internal Transcribed Spacer-1 rRNA region was used to identify and quantify Old World *Leishmania* in 300 samples from human patients, reservoir hosts and sand flies. Different characteristic high resolution melt analysis patterns were exhibited by *L. major*, *L. tropica*, *L. aethiopica*, and *L. infantum*. Genotyping by high resolution melt analysis was verified by DNA sequencing or restriction fragment length polymorphism. This new assay was able to detect as little as 2-4 ITS1 gene copies in a 5 µl DNA sample, i.e., less than a single parasite per reaction.

**Conclusions/Significance:**

This new technique is useful for rapid diagnosis of leishmaniasis and simultaneous identification and quantification of the infecting *Leishmania* species. It can be used for diagnostic purposes directly from clinical samples, as well as epidemiological studies, reservoir host investigations and vector surveys.

## Author Summary

Protozoal parasites of the genus *Leishmania* are transmitted by sand fly bites to humans and animals. Three major forms of disease are caused by these parasites: cutaneous leishmaniasis, responsible for disfiguring skin wounds; mucocutaneous leishmaniasis, causing non-healing ulceration around the mouth and nose; and the potentially fatal visceral leishmaniasis, involving internal organs such as the spleen and liver. More than 2 million new human infections are caused annually by leishmaniasis globally, it is endemic in more than 88 countries and prevalent also as an imported disease in non-endemic regions due to travel and tourism. Most species of *Leishmania* that infect humans are zoonotic and transmitted from animal reservoir hosts. As various leishmanial parasites cause disease with similar symptoms, but require different therapeutic regimens and have dissimilar prognoses, reliable, sensitive and rapid diagnostic assays are needed. This study focuses on the five main species that cause leishmaniasis in the Old World. It presents a new assay for rapid detection, species identification and quantification of leishmanial parasites in clinical samples, reservoir hosts and sand flies. This technique could be especially valuable in regions where several leishmanial species exist, in non-endemic regions where infected patients require a rapid diagnosis, and for epidemiological host and vector studies leading to prevention programs.

## Introduction

Molecular methods are increasingly employed for diagnostic and epidemiological studies on leishmaniasis in an effort to detect infection and categorize *Leishmania* at the genus, species or strain level [1]. Ideal assays should be easy to perform and interpret, rapid, sensitive, specific, and able to determine parasite loads accurately in hosts and vectors. Several techniques have been described for the identification and characterization of *Leishmania* at the molecular level. These include PCR- restriction fragment length polymorphism (RFLP), sequence analysis of multicopy genes and intergenic spacer regions, DNA fingerprinting and randomly amplified polymorphic DNA, and PCR followed by reverse line blot hybridization [1]–[4]. Multilocus enzyme electrophoresis is an additional characterization technique that relies on variation in *Leishmania* enzymes electrophoretic mobility [5]. Several of these techniques, such as multilocus enzyme electrophoresis and randomly amplified polymorphic DNA, require isolation and culture of parasites, limiting their use in clinical situations where rapid diagnosis is required [1].

Three major forms of human disease are encountered: cutaneous leishmaniasis, visceral leishmaniasis and mucocutaneous leishmaniasis. In the Old World, the two major forms are cutaneous and visceral leishmaniasis, and the main parasite species responsible for these diseases are *Leishmania infantum* (visceral and cutaneous leishmaniasis), *L. donovani* (visceral leishmaniasis), *L. tropica* (cutaneous leishmaniasis), *L. aethiopica* (cutaneous and mucocutaneous leishmaniasis) and *L. major* (cutaneous leishmaniasis). Accurate and sensitive diagnostic and identification procedures are required to distinguish between these species, whose geographic distribution frequently overlaps, to enable adequate treatment and appropriate public health control measures.

High resolution melt analysis (HRM) is an automated analytical molecular technique that measures the rate of double stranded DNA dissociation to single stranded DNA with increasing temperature. This dissociation is monitored by including a fluorescent dye in the PCR reaction that intercalates homogenously into DNA, and only fluoresces, when bound to dsDNA. The change in fluorescence measures the thermally-induced DNA dissociation by HRM and the observed melting behavior is characteristic of the particular DNA product as determined based on sequence length, GC content, complimentarity, and nearest neighbor thermodynamics. HRM has been used for the detection of single nucleotide polymorphism in genetic diseases. It was subsequently used for detection of internal tandem duplications, simultaneous mutation scanning and genotyping in bacteriology, cancer research and hematology [6],[7]. It is a sensitive technique readily applied to pathogen detection, using DNA extracted directly from blood and other tissues, eliminating lengthy procedures such as parasite isolation and growth. Results are obtained without additional post-PCR processing in <2.5 hrs. We describe a new application of HRM for the rapid detection, quantification and speciation of Old World leishmanial species.

## Materials and Methods

Samples were obtained from humans and domestic dogs as part of routine diagnosis of leishmaniasis, and from wild animals and sand flies during epidemiological studies. The study that concerned animals was conducted adhering to the Hebrew University's guidelines for animal husbandry and use of animals in research. The use of patient samples was approved by the Helsinki Committee for Human Research of the Hadassah Hospital, Ein Kerem, Jerusalem. Since the study was a part of routine diagnosis of suspected leishmaniasis and the diagnostic samples were submitted from several distant health care facilities, only oral informed consent was required and obtained. Informed consent was recorded in writing in the patient's file as required by the IRB committee.

A total of 300 samples were examined, 159 from human leishmaniasis patients, 78 from naturally infected dogs, 38 from hyraxes, 15 from rodent species (*Psammomys obesus*, *Apodemus mystacinus*, *Gerbillus dasyurus*, *Acomys cahirinus*, *Meriones sacramenti* and *Eliomus melanurus*) and 10 from sand flies (*Ph. arabicus*, *Ph. paptasi*, *Ph. rossi* and *Ph. sergenti*). A hundred and seventy one samples were from the Middle East (Iran, Iraq, Israel, Jordan, the Palestinian Authority, Saudi Arabia and Turkey), 82 from Asia (Afghanistan, Azerbaijan, China, Georgia, India, Turkmenistan, and Uzbekistan), 35 from Africa (Algeria, Ethiopia, Kenya, Morocco, Namibia, Senegal, Sudan, and Tunisia) and 12 from Europe (Greece, Italy, Portugal and Spain). Of these, 131 DNA samples were purified from cultured promastigotes isolated from 98 humans, 21 dogs, 10 sand flies, 1 hyrax and 1 rodent. The remaining DNA samples were extracted directly from tissues including: human cutaneous lesions (66), blood (dogs-47 and hyraxes-12), skin biopsies (rodents-14 and hyraxes-16), spleens (hyraxes-4 and dogs-10) and lymph nodes (dogs-10). DNA was extracted from cultured parasites and directly from parasites in tissue smears made from cutaneous lesions, blood, skin or spleen biopsies by either the phenol-chloroform or guanidine thio-cyanate techniques [4],[8].

A 265–288 bp fragment, depending on the leishmanial species, within the internal transcribed spacer 1 (ITS1) region of the leishmanial ribosomal RNA operon was amplified by real-time PCR using the primers ITS-219F (5′- AGCTGGATCATTTTCCGATG- 3′) and ITS-219R (5′- ATCGCGACACGTTATGTGAG) designed for this study and then examined by HRM analysis. *Leishmania* ITS1 DNA sequences were compared by multi-alignment (ClustalW2; http://www.ebi.ac.uk/Tools/clustalw2/index.html). Genomic DNA from all *Leishmania* strains except for one taken from GenBank (MHOM/ET/1972/L102) were verified by partial sequencing of the ITS1 at the Center for Genomic Technologies, Hebrew University of Jerusalem.

The assay's specificity was evaluated with the following non-leishmanial trypnosomatids: *Crithidia fasciculata* (ATCC 11745), *Leptomonas seymouri* (ATCC 30220), *Trypanosoma brucei* (BF 427), *T. evansi* (RoTot 1.2), *T. cruzi*, and *T. equinum. Trypanososma cruzi and T. equinum* DNA were kindly supplied by Dr. P. Michels and Dr. V. Hannaert from the Université Catholique de Louvain, Belgium. DNA from control non-infected humans, dogs, hyraxes and rodents were also evaluated for possible response with the HRM PCR.

The PCR reaction was performed in a total volume of 20 µl containing 5 µl DNA, 40 nM of each primer, 10 µl Thermo-start PCR Master Mix (Thermo-start ABgene, Rochester New York USA), 0.6 µl 100-fold diluted SYTO9 (Invitrogen, Carlsbad, CA), and sterile, DNase/RNase-free water (Sigma, St. Louis, USA) using a Rotor-Gene 6000 real-time PCR machine (Corbett Life Science). Initial denaturation for 15 min at 95°C was followed by 40 cycles of denaturation at 5 sec at 95°C per cycle, annealing and extension for 30 sec at 57°C, and final extension for 1 sec at 76°C. This was followed by a conventional melting step from 60 to 95°C at 1°C/sec, after which the temperature was slowly decreased from 90 to 50°C (1°C/sec) to allow re-annealing. In the final step, HRM analysis was carried out increasing the temperature from 75 to 90°C at 0.4°C/sec increments. All samples were examined in duplicate.

A set of control DNA standards from cultured promastigotes were prepared and analyzed in parallel with the test samples to standardize the real-time PCR. Promastigotes of *L. infantum* were suspended in un-infected human blood counted in a counting chamber (Z2, Beckman Coulter, Inc) to give a concentration of 5×10^7^ parasites/ml. Ten microliters (5×10^5^ parasites) were suspended in 90 µl of un-infected human blood giving 5×10^3^ parasites/µl. Samples (10 ul) were then diluted tenfold six times and the DNA was extracted with guanidine thio-cyanate followed by silica beads [8]. Purified DNA was suspended in 100 µl of elution buffer and serially diluted from 5×10^2^ to 5×10^−2^ parasites/µl. Five µl of DNA were used for each reaction.

Restriction fragment length polymorphism (RFLP) was performed according to Schonian et al., 2003 [9]. DNA sequences were compared for similarity to sequences in GenBank using the BLAST program hosted by NCBI, National Institutes of Health, USA (http://www.ncbi.nlm.nih.gov).

## Results

DNA sequence multi-alignment analysis of nine strains from five Old World *Leishmania* species for the ITS1 regions amplified by the primers used in this study indicated ≥82% similarity between the sequences (Figure S1). The size for the product amplified ranged from 265 bp (*L. infantum* and *L. donovani*) to 288 bp (*L. aethiopica*). Strains from different foci were used in order to account for potential variation in the ITS1 sequence among *Leishmania* species originating from a wide range of geographical locations. For example, *L. donovani* from India as well as an African strain were used, and *L. tropica* isolates from Tiberias and the Northern Sea of Galilee in Israel were used due to the known interspecies variation between them [10]. Altogether 30 different mismatch stretches spanning from 1 to 18 nucleotides each were observed. Longer sequence deletions, between nucleotides 172–177 and 236–253, created gaps in alignment between several species. Insertion/deletion mismatches at positions 47–48; 62; 100–101 allowed the discrimination of *L. tropica* and *L. aethiopica* from *L. major, L. infantum* and *L. donovani*. Insertion/deletion mismatches at position 66–68; 162–163, 184–185; 215; 242–250 and 267–268 positions separated *L. infantum* and *L. donovani* from the other three species. A deletion at position 142–143 and an insertion at position 162–164 separated *L. major* from the other species. Insertion of 6 bases between positions 172–177 and a deletion between positions 248–253 separated *L. aethiopica* from all other species. Other nucleotide mismatches such as transversions and transitions were also present, for example within *L. tropica* thus separating the strains of the North Sea of Galilee from the Tiberias and central Israel ones. Differences between *L. tropica* from these areas included deletions at the 228 and 241 positions, deletion/insertion at the 242–244 positions, three transitions and a number of transversions. The African *L. donovani* strain was different from the Indian strain by transversions at the 46, 57, and 80–82 positions.

Leishmanial DNA was amplified and analyzed by HRM PCR from 300 samples. Species identification was confirmed by RFLP for 156 samples, by DNA sequencing of the ITS1 PCR products for165 samples, and by both techniques for 21 samples. The species identified in the samples were *L. infantum* (n = 143), *L. tropica* (n = 86), *L. major* (n = 52), *L. aethiopica* (n = 7) and *L. donovani* (n = 12).

The real-time PCR standard curve was linear (R^2^ = 0.998) over a 5-log range of DNA concentrations and showed a 110% reaction efficiency as determined from the slope (−3.1) of the curve (http://www.stratagene.com/techtoolbox/calc/qpcr_slope_eff.aspx). No amplification was noted at the dilutions of 5×10^−3^ and 5×10^−4^, and therefore 5×10^−2^ marked the lowest dilution where parasite DNA was detected. The lower limit of sensitivity from the standard curve was determined as 0.25 parasites per sample.

**Figure 1:**
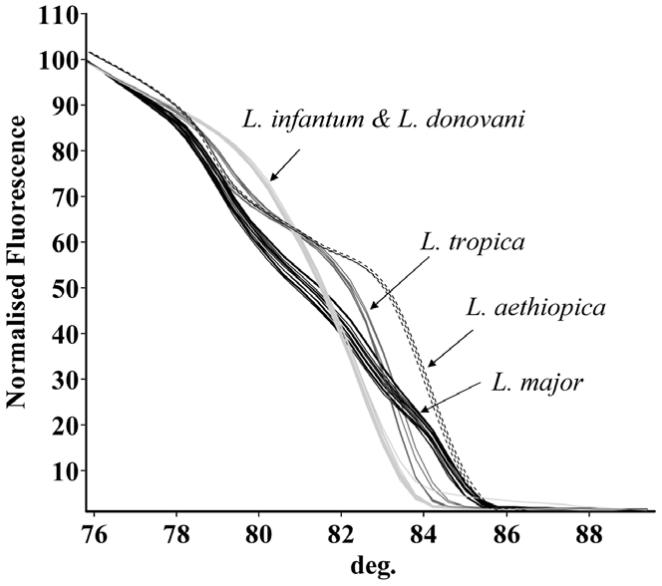
High resolution melting curves. High resolution melting (HRM) curves of the 265–288 bp ITS1-PCR amplicon of Old World *Leishmania* species. Normalized fluorescence is plotted against degrees C° (deg.). The curves include parasites from different hosts and geographic origins including 7 strains of *L. major*, 5 of *L. aethiopica*, 7 of *L. tropica*, 13 of *L. infantum* and 2 of *L. donovani*.

The normalized HRM curves for the amplicons from the five leishmanial species are shown in Figure 1. Each *Leishmania* species produced a unique melting plot that was easily distinguishable from other species and consistent with the observed nucleotide differences among them, except for *L. infantum* that shared a plot similar to *L. donovani*. HRM analysis showed very uniform patterns for every species compared to the corresponding conventional melting curves, highlighting the differences between the species and reducing misidentification. Melting curve patterns were consistent for each species whether the DNA sample was from cultured promastigotes, or amastigotes in blood and tissue samples. Non-template controls (NTC) showed no signal after 40 amplification cycles and primer-dimer formation was not noted. The normalization regions used for the analysis ranged from 75.23°C to 75.75°C in the leading range, and from 89.4°C to 89.8°C in the trailing range. Complete agreement was found between speciation by HRM analysis, RFLP and/or DNA sequencing.

**Figure 2:**
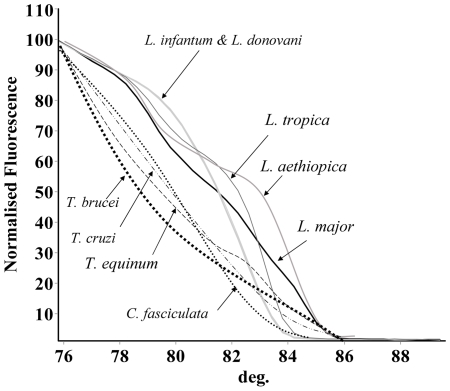
High resolution melting curves of Old World *Leishmania* species compared with non-leishmanial trypanosomatids. High resolution melting (HRM) curves for the ITS1-PCR amplicon from Old World *Leishmania* species [*L. major* (MHOM/TM/1973/5ASKH), *L. infantum* (MHOM/TN/1980/IPT1), *L. donovani* (MHOM/IN/1980/DD8), *L. tropica* (ISER/IL/2002/LRC-L909) and *L. aethiopica* (MHOM/ET/1972/L102)] and the non-leishmanial trypanosomatids *Trypanosoma brucei*, *T. cruzi*, *T. equinum* and *Crithidia fasciculata*. Normalized fluorescence is plotted against degrees C° (deg.).

HRM PCR specificity was examined using DNA from other trypanosomatids. PCR products were observed with *T. brucei*, *T. cruzi*, *T. equinum* and *C. fasciulata* but not *L. seymouri* and *T. evansi* (Figure 2). However, the amplicons produced using the non-leishmanial DNAs were larger and the corresponding HRM plots were markedly different and did not overlap with any of the *Leishmania* spp. (Figure 2). Host DNA from non-infected humans, dogs, hyraxes and rodents was not amplified and no HRM plot was observed with this assay.

Though unique identifying HRM patterns were discerned for each Old World *Leishmania* species, slight differences within a species were noticed among strains that coincided with specific leishmanial genotypes and/or geographic distribution, and could be correlated with nucleotide substitutions, insertions and/or deletions in the real-time PCR product. For instance, *L. tropica* strains from Israel originating just north of the Sea of Galilee (microsatellite clade IV genotype) and those from the vicinity of Tiberias, central and southern Israel (microsatellite clade I genotype) have been shown to be different by several molecular and biological criteria, including cell-surface antigens and DNA microsatellite analyses [10],[11]. Strains from these adjacent regions were easily recognized since they gave slightly different HRM patterns due to differences in nucleotide sequence within the ITS1 fragment amplified in this assay (Figures S1 and 3).

**Figure 3:**
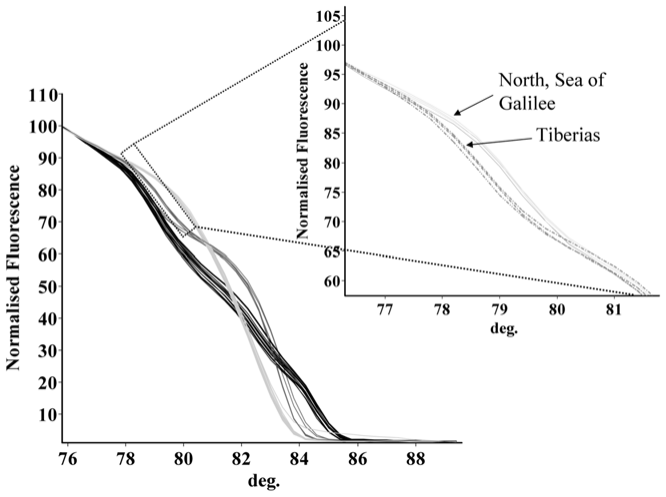
High resolution melting curves of *Leishmania tropica* strains belonging to two microsatellite clusters in different regions of Israel. Main part of the figure shows HRM plots of *Leishmania* species found in Israel (*L. major*, *L. infantum* and *L. tropica*). Insert is a magnification of the HRM region allowing differentiation between *L. tropica* strains belonging to microsatellite cluster IV (North Sea of Galilee) and microsatellite cluster I (central and southern Israel).

## Discussion

This study describes a new technique for the rapid detection, quantification and species identification of Old World leishmanial species. HRM analysis is sensitive and can detect as little as 2–4 ITS1 gene copies in a 5 µl DNA sample, i.e., less than a single parasite per reaction. It is a closed tube assay that does not employ additional fluorescent probes and simply utilizes a DNA melting assay and computerized analysis of the results to produce a graphic output, thus the risk of contamination of the samples is decreased. HRM analysis provides a distinct characteristic repeatable melting curve for each species that retains its general shape even when strains are from distant locations and have different hosts and vectors. It distinguished all the Old World leishmanial species causing human disease, except *L. infantum* from *L. dononvani.* These two species gave similar HRM curves. As the DNA sequence over the 265 bp region amplified by this PCR is almost identical for *L. infantum* and *L. donovani*, this finding is not surprising. Both species cause visceral leishmaniasis, and prognosis and treatment of this disease is similar. Development of an HRM assay that separates between *L. infantum* and *L. donovani* should be possible by choosing a short DNA region unique for each species, since this technique can potentially distinguish between DNA sequences that differ by only a single nucleotide. The technique was also able to discern inter-species variation as seen with strains of *L. tropica* isolates from the two Israeli foci.

The appearance of complex HRM plots showing shoulders for the species giving larger PCR products such as *L. tropica* and *L. aethiopica*, is likely due to the presence of multiple melting domains with different Tm within the dsDNA amplicons. The non-leishmanial trypnosdomatids that were amplified by the reaction produced distinctly different curves that presented in an area of the plot situated away from the *Leishmania* spp. and not overlapping with them. Therefore, despite the fact that the assay amplifies the DNA of some other trypnosomatids, they can not be confused with the *Leishmania* spp. evaluated.

Real-time PCR has been used for previous studies on different aspects of leishmaniasis including diagnosis, animal models, drug efficacy and vectorial capacity [12]–[17]. Different target genes and loci have been used including the *Leishmania* DNA polymerase gene [12], kinetoplast DNA [13],[15],[16] and the SSU rRNA gene [14],[17]. ITS1 HRM analysis further simplifies disease diagnosis by allowing rapid parasite identification and quantification, within less than 2.5 hrs, without need for species or genus specific probes. Furthermore, we have shown that HRM can be used for direct testing of patient samples such as skin smear, skin biopsy, visceral organ tissues and blood, and can be used for diagnostic purposes as well as epidemiological studies, reservoir host investigations and vector surveys. This technique could be especially valuable in regions where several leishmanial species exist causing disease with similar symptoms, but requiring different treatment regimens and having dissimilar prognosis. *Leishmania major*, *L. tropica* and *L. infantum* overlap in the Middle East and North Africa. In Israel, *L. major* and *L. tropica* cause a similar cutaneous disease whereas in Morocco, the situation is more complicated with cutaneous leishmaniasis caused by these two species and also by *L. infantum* [18],[19]. This assay would also be useful in medical centers in non-endemic regions where infected patients require a rapid diagnosis at the *Leishmania* species level to receive correct therapy and prognostication. Its ability to quantify infection would make it useful in evaluating the success of therapy and new types of treatments in experimental animals and in tissue and cell culture systems.

## Supporting Information

Figure S1 Alignments of ITS1 sequences. Multiple alignment of nine *Leishmania* strains including *L. infantum* (MHOM/TN/1980/IPT1, MHOM/ES/1993/PM1); *L. donovani* [MHOM/IN/1980/DD8, MHOM/ET/1967/HU3 (LV9)]; *L. major* (MHOM/TM/1973/5ASKH, MHOM/SN/1996DPPE23); *L. tropica* (ISER/IL/1998/LRC-L758 and ISER/IL/2002/LRC-909); and *L. aethiopica* (MHOM/ET/1972/L102). The dark grey areas in the 5′- and 3′-ends of the sequences represent the oligonucleotide primers used for amplification. The light gray areas represent nucleotide mismatches between the aligned sequences.
(0.01 MB PDF)
