# Supplementary material for: Detection and Identification of Old World Leishmania by High Resolution Melt Analysis
Source: PLoS Negl Trop Dis. 2010 Jan 12;4(1):e581. doi: 10.1371/journal.pntd.0000581 (PMC2797090; doi:10.1371/journal.pntd.0000581)
Supplement: Figure S1 — Alignments of ITS1 sequences. Multiple alignment of nine Leishmania strains including L. infantum (MHOM/TN/1980/IPT1, MHOM/ES/1993/PM1); L. donovani [MHOM/IN/1980/DD8, MHOM/ET/1967/HU3 (LV9)]; L. major (MHOM/TM/1973/5ASKH, MHOM/SN/1996DPPE23); L. tropica (ISER/IL/1998/LRC-L758 and ISER/IL/2002/LRC-909); and L. aethiopica (MHOM/ET/1972/L102). The dark grey areas in the 5′- and 3′-ends of the sequences represent the oligonucleotide primers used for amplification. The light gray areas represent nucleotide mismatches between the aligned sequences. (0.01 MB PDF) [file pntd.0000581.s001.pdf]

```
1)  L. infantum                          AGCTGGATCATTTTCCGATGATTACACCCAAAAAAAACATATACAA--CT 50
2)  L. infantum                          AGCTGGATCATTTTCCGATGATTACACCCAAAAAAAACATATACAA--CT 50
3)  L. donovani                          AGCTGGATCATTTTCCGATGATTACACCCAAAAAAAACATATACAA--CT 50
4)  L. donovani                          AGCTGGATCATTTTCCGATGATTACACCCAAAAAAAACATATACAC--CT 50
5)  L. major                             AGCTGGATCATTTTCCGATGATTACACCCAAAAAAAACATATACAA--CT 50
6)  L. major                             AGCTGGATCATTTTCCGATGATTACACCCAAAAAAAACATATACAA--CT 50
7)  L. tropica                           AGCTGGATCATTTTCCGATGATTACACCCAAAAAAAACATATACAAAACT 50
8)  L. tropica                           AGCTGGATCATTTTCCGATGATTACACCCAAAAAAAACATATACAAAACT 50
9)  L. aethiopica                        AGCTGGATCATTTTCCGATGATTACACCCAAAAAAAACATATACAAAACT 50
                                         ********************************************    **

1)  MHOM/TN/1980/IPT1                    CGGGGAAGACC-TAT---GTATATATATAGTGTAGGCCTTTCCCACATA- 100
2)  MHOM/ES/1993/PM1                     CGGGGAAGACC-TAT---GTATATATATAGTGTAGGCCTTTCCCACATA- 100
3)  MHOM/IN/1980/DD8                     CGGGGAAGACC-TAT---GTATATATATAGTGTAGGCCTTTCCCACATA- 100
4)  MHOM/ET/1967/HU3(LV9)                CGGGGAGGACC-TAT---GTATATATATATGTTAGGCCTTTCCCACATA- 100
5)  MHOM/TM/1973/5ASKH                   CGGGGAAGGCT-TATTCTATATATATATAGTATAGGCTTTTCCCACATA- 100
6)  MHOM/SN/1996/DPPE23                  CGGGGAAGGCT-TATTCTATATATATATAGTATAGGCTTTTCCCACATA- 100
7)  ISER/IL/2002/LRC-L909                CGGGGAGGCCTATATATTATACATTATTATATAGGCCTTT-CCCACCCTT 100
8)  ISER/IL/1998/LRC-L758                CGGGGAGGCCTATATATTATACATTATTATATAGGCCTTT-CCCACACAT 100
9)  MHOM/ET/1972/L102                    CGGGGCAGGCGCTATTATATATAT-TATTATAGGACCCTTTCCCACATAC 100
                                         *****  * *  ***    ** **            **** *****

1)  MHOM/TN/1980/IPT1                    -CACAGCAAAGTTTTGTACTCAAAATTTGCAGTAAAAAAAAGGCCGATC 150
2)  MHOM/ES/1993/PM1                     -CACAGCAAAGTTTTGTACTCAAAATTTGCAGTAAAAAAAAGGCCGATC 150
3)  MHOM/IN/1980/DD8                     -CACAGCAAAGTTTTGTACTCAAAATTTGCAGTAAAAAAAAGGCCGATC 150
4)  MHOM/ET/1967/HU3(LV9)                -CACAGCAAAGTTTTGTACTCAAAATTTGCAGTAAAAAAAAGGCCGATC 150
5)  MHOM/TM/1973/5ASKH                   -CACAGCAAACTTTTATACTCAAAATTTGCAGTAAAAAAG--GCCGATC 150
6)  MHOM/SN/1996/DPPE23                  -CACAGCAAACTTTTATACTCAAAATTTGCAGTAAAAAAG--GCCGATC 150
7)  ISER/IL/2002/LRC-L909                CCCCGGCAAACTTTTATACTCAAGTTTGGCAGTAAACAAAAGGCCGATC 150
8)  ISER/IL/1998/LRC-L758                ACACAGCAAACTTTTATACTCGAAGTTTGCAGTAAACAAAAGGCCGATC 150
9)  MHOM/ET/1972/L102                    ACACAGCAAACTTTTATACTCGAAGTTTGCAGTAAAGAAAAGGCCGATC 150
                                                *** **** ***** *  ** ******** **   *******

1)  MHOM/TN/1980/IPT1                    GACGTTATAAC--GCACCGCC------TATACA--AAAGCAAAAATGTCC 200
2)  MHOM/ES/1993/PM1                     GACGTTATAAC--GCACCGCC------TATACA--AAAGCAAAAATGTCC 200
3)  MHOM/IN/1980/DD8                     GACGTTATAAC--GCACCGCC------TATACA--AAAGCAAAAATGTCC 200
4)  MHOM/ET/1967/HU3(LV9)                GACGTTATAAC--GCACCGCC------TATACA--AAAGCAAAAATGTCC 200
5)  MHOM/TM/1973/5ASKH                   GACGTTGTAGAACGCACCGCC------TATACACAAAGCAAAAATGTCC 200
6)  MHOM/SN/1996/DPPE23                  GACGTTGTAGAACGCACCGCC------TATACACAAAGCAAAAATGTCC 200
7)  ISER/IL/2002/LRC-L909                GACGTTTTACCC--CACCGCC      TATCCCCAAAGCAAAAATGTCC 200
8)  ISER/IL/1998/LRC-L758                GACGTTATAACG——CACCGCC      TATACACAAAGCAAAAATGTCC 200
9)  MHOM/ET/1972/L102                    GACGTTATATAACGCACCGCCCGCCTATATACACAAAGCAAAAATGTCC 200
                                         ****** **     *******      ***     ***************

1)  MHOM/TN/1980/IPT1                    GTTTATACAAAAAA-TATACGGCGTTTCGGTTTTTGGCGGG--------- 250
2)  MHOM/ES/1993/PM1                     GTTTATACAAAAAA-TATACGGCGTTTCGGTTTTTGGCGGG--------- 250
3)  MHOM/IN/1980/DD8                     GTTTATACAAAAAA-TATACGGCGTTTCGGTTTTTGGCGGG--------- 250
4)  MHOM/ET/1967/HU3(LV9)                GTTTATACAAAAAA-TATACGGCGTTTCGGTTTTTGGCGGG--------- 250
5)  MHOM/TM/1973/5ASKH                   GTTTATACAAAAAAATAGACGGCGTTTCGGTTTTTGGCGGGAGGGAGAGA 250
6)  MHOM/SN/1996/DPPE23                  GTTTATACAAAAAAATAGACGGCGTTTCGGTTTTTGGCGGGAGGGAGAGA 250
7)  ISER/IL/2002/LRC-L909                GTTTATCCAAATTTA----CCGGCTTTCCGGTTTTGTTGGCGGG------ 250
8)  ISER/IL/1998/LRC-L758                GTTTATACAAATATA----CGGCGTTTC-GGTTTTGTTGG---------- 250
9)  MHOM/ET/1972/L102                    GTTTATACAAATATA----CGGCGTTTCGGTTTTTTGTTGGCGGGGG--- 250
                                         ****** ****        * *  ***    ****

1)  MHOM/TN/1980/IPT1                    ---GTGGGTGCGTGTG--TGGATAACGGCTCACATAACGTGTCGCGAT 298
2)  MHOM/ES/1993/PM1                     ---GTGGGTGCGTGTG--TGGATAACGGCTCACATAACGTGTCGCGAT 298
3)  MHOM/IN/1980/DD8                     ---GTGGGTGCGTGTG--TGGATAACGGCTCACATAACGTGTCGCGAT 298
4)  MHOM/ET/1967/HU3(LV9)                ---GTGGGTGCGTGTG--TGGATAACGGCTCACATAACGTGTCGCGAT 298
5)  MHOM/TM/1973/5ASKH                   GAGGGGGGTGCGTGTGCGTGGATAACGGCTCACATAACGTGTCGCGAT 298
6)  MHOM/SN/1996/DPPE23                  GAGGGGGGTGCGTGTGCGTGGATAACGGCTCACATAACGTGTCGCGAT 298
7)  ISER/IL/2002/LRC-L909                GGGGGGGTCCTTTGTGTGTGGATAACGGCTCACATAACGTGTCGCGAT 298
8)  ISER/IL/1998/LRC-L758                CGGGGGGTGGCGTGTGTGTGGATAACGGCTCACATAACGTGTCGCGAT 298
9)  MHOM/ET/1972/L102                    ---GGGGGTGCGTGTGTGTGGATAACGGCTCACATAACGTGTCGCGAT 298
                                            * **     ****  ******************************
```
